# Root aeration improves growth and nitrogen accumulation in rice seedlings under low nitrogen

**DOI:** 10.1093/aobpla/plv131

**Published:** 2015-11-17

**Authors:** Jingwen Zhu, Jing Liang, Zhihui Xu, Xiaorong Fan, Quansuo Zhou, Qirong Shen, Guohua Xu

**Affiliations:** 1 State Key Laboratory of Crop Genetics and Germplasm Enhancement, Nanjing Agricultural University, Nanjing, Jiangsu 219500, China; 2 Key Laboratory of Plant Nutrition and Fertilization in Lower-Middle Reaches of the Yangtze River, Ministry of Agriculture, Nanjing Agricultural University, Nanjing, Jiangsu 219500, China

**Keywords:** Aerenchyma formation, ethylene, gene expression, nitrogen, *Oryza sativa*, oxygen

## Abstract

In wetland soils, changes in oxygen (O_2_) level in the rhizosphere are believed to influence the behaviour of nutrients and their usage by plants. However, the effect of aeration on nitrogen (N) acquisition under different N supply conditions remains largely unknown. In this study, the rice cultivars Yangdao 6 (YD6, with higher root aerenchyma abundance) and Nongken 57 (NK57, with lower root aerenchyma abundance) were used to evaluate the effects of aeration on rice growth and N accumulation. Our results showed that the number of adventitious roots and the root surface area increased significantly, and ethylene production and aerenchyma formation decreased in both cultivars after external aeration (EA). Five N treatments, including no N (−N), 0.125 mM NH_4_NO_3_ (LN), 1.25 mM Ca(NO_3_)_2_ (NO_3_-N), 1.25 mM (NH_4_)_2_SO_4_ (NH_4_-N) and 1.25 mM NH_4_NO_3_ (N/N), were applied to YD6 and NK57 for 2 days under internal aeration or EA conditions. External aeration increased the root biomass in both cultivars and the shoot biomass in NK57 by 18–50 %. The total N concentrations in roots of YD6 grown under −N and LN and of NK57 grown under NO_3_-N were increased by EA. Expression of *OsPAD4*, one of four putative genes regulating aerenchyma formation, showed a similar pattern alongside changes in the ethylene level in the EA-treated rice irrespective of the N treatments. Furthermore, expression of the high-affinity nitrate transporter gene *OsNRT2.1* was increased by EA under −N, LN and NO_3_-N conditions. Our data provide evidence of an interaction between O_2_ and the supply of N in ethylene production, aerenchyma formation and N nutrition through modification of the expression of *OsPAD4* and *OsNRT2.1*.

## Abstract (precis)

As rice adapts to waterlogged conditions it develops aerenchyma to transport O_2_ from shoots to roots and leaches O_2_ into the rhizosphere to balance the soil redox potential for rice root growth and the growth of bacteria such as ammonium oxidation bacteria, which can convert ammonium into nitrate and influence the total N uptake by rice roots. We showed that rice cultivars differ in aerenchyma development, nitrogen uptake and gene response to external aeration treatment. Interestingly, we found that the effect of nitrogen on rice plant growth depends on environmental O_2_ level.

## Introduction

Wetland plants commonly grow under alternating drought and flood conditions (Alberda 1953; Colmer 2003*a*; Qian *et al*. 2004; Nishiuchi *et al*. 2012). They can develop aerenchyma—gas-filled interconnected spaces or lacunae in the roots, stems and leaves—to adapt to low dissolved oxygen (O_2_) levels during waterlogging (Arenovski and Howes 1992; Colmer *et al*. 1998; Armstrong and Armstrong 2005; Steffens *et al*. 2011). Oxygen, ethylene and methane can be transported from the shoots to the roots via the aerenchyma and released into the rhizosphere (Armstrong 1979; Jackson and Armstrong 1999; Colmer 2003*b*; Teal and Kanwisher 1966; Yu *et al*. 1997). This leads to variable dissolved O_2_ levels at wetland plant root surfaces in paddy fields. Oxygen directly or indirectly affects nitrification dominated by nitrifying bacteria, or chemical oxidation for the conversion of $\text{N}{{\text{H}}_{4}}^{+}$ to $\text{N}{{\text{O}}_{3}}^{-}$ at the root surface, as well as the form of nitrogen (N) taken up by wetland plants (Kludze *et al*. 1993; Li *et al*. 2008). Oxygen levels and rate of nitrification in the paddy rhizosphere can also affect total N usage by wetland plants (Li *et al*. 2008; Xu *et al*. 2012*a*).

Aerenchyma form in the parenchyma as a result of programmed cell death (PCD) and extend from below the ground up through the stems and leaves (Drew *et al*. 2000). Formation of aerenchyma is affected by external ethylene but to different extents among different cultivars (Justin and Armstrong 1991; Kong *et al*. 2009). In plant roots, external ethylene can inhibit root elongation (Visser and Pierik 2007) and aerenchyma formation (Takahashi *et al*. 2015). In *Arabidopsis*, the formation of lysigenous aerenchyma as a result of PCD is dependent on the plant defence regulators *AtLSD1*, *AtEDS1* and *AtPAD4* (Mühlenbock *et al*. 2007). These genes influence PCD by operating upstream of ethylene production and reactive oxygen species in the roots (Mühlenbock *et al*. 2007). Expression of *PAD4* was shown to be strongly associated with ethylene concentrations in *Arabidopsis* (Durrant and Dong 2004; Lei *et al*. 2014) and rice (Qiu *et al*. 2007; Ding *et al*. 2008). In rice, a gene related to disease resistance, *OsBphi008a*, was located downstream of the ethylene signalling pathway and was a positive indicator of ethylene levels (Yuan *et al*. 2004; Hu *et al*. 2011), and overexpression of *OsPDCD5* induced PCD in transgenic lines (Su *et al*. 2006). In addition, *OsLSD1.1*, *OsLSD2*, *OsPAD4* and *OsEDS* in rice, which are homologous to the *Arabidopsis AtLSD1*, *AtPAD4* and *AtEDS1* genes, respectively, may also play similar roles during aerenchyma formation via PCD in the roots.

Although ammonium $(N{{\text{H}}_{4}}^{+})$ is the primary form of available N in flooded fields, and rice prefers $\text{N}{{\text{H}}_{4}}^{+}$ over $\text{N}{{\text{O}}_{3}}^{-}$ (Wang *et al.* 1993), physiological studies have shown that lowland rice is exceptionally efficient at acquiring $\text{N}{{\text{O}}_{3}}^{-}$ through nitrification in the rhizosphere (Li *et al*. 2006; Duan *et al*. 2007). A mixed supply of $\text{N}{{\text{H}}_{4}}^{+}$ and $\text{N}{{\text{O}}_{3}}^{-}$ to both upland and lowland rice cultivars resulted in significant increases in the dry weight and grain yield compared with application of either $\text{N}{{\text{H}}_{4}}^{+}$ or $\text{N}{{\text{O}}_{3}}^{-}$ as the sole N source (Qian *et al*. 2004; Duan *et al*. 2007). In rice, a number of ammonium transporter genes (Sonoda *et al*. 2003; Zhao *et al*. 2014) and nitrate transporter genes (Feng *et al*. 2011; Xu *et al*. 2012*b*) have been functionally characterized. The ammonium transporter genes *OsAMT1.1*, *OsAMT1.2* and *OsAMT1.3* exhibited ammonium transport activity in yeast (Sonoda *et al*. 2003) and *OsAMT1.1* contributed more than did the other two genes to N accumulation (Zhao *et al*. 2014). Knockdown of *OsNAR2.1*, which encodes a partner protein of the NRT2 transporter, decreased the total N accumulation by ∼63–66 % (Yan *et al*. 2011). OsNRT2.3a plays a key role in long-distance $\text{N}{{\text{O}}_{3}}^{-}$ transport from the roots to shoots in rice under low $\text{N}{{\text{O}}_{3}}^{-}$ conditions (Tang *et al*. 2012). Physiological studies on the relationship between N and the aerenchyma in rice have shown that the aerenchyma can influence N absorption and utilization (Yoshida and Ancajas 1973; Gebauer *et al*. 1996; Li *et al*. 2008). In addition, the different forms of N could also affect aerenchyma formation. The $\text{N}{{\text{O}}_{3}}^{-}$ treatment yielded a significantly greater increase in root porosity than did $\text{N}{{\text{H}}_{4}}^{+}$ treatment (Yang *et al*. 2012), and N deficiency induced aerenchyma formation in the roots of maize (Drew *et al*. 1989; He *et al*. 1994) and rice (He *et al*. 1992).

Although the physiological effects of N treatments on aerenchyma formation have been investigated in plants, limited information is available on the underlying molecular mechanisms. In previous research, we characterized two rice cultivars with high (Yangdao 6, YD6) and low (Nongken 57, NK57) N use efficiency (NUE), exhibiting different ammonia-oxidizing bacteria and nitrification activities in the root rhizosphere (Li *et al*. 2008). We reported the effects of root aeration on root aerenchyma formation, N accumulation and the expression of aerenchyma-formation-related and N transporter genes in both cultivars under diverse N conditions after internal aeration (IA) and external aeration (EA) treatments. These results provided evidence for the relationship between aerenchyma formation and N nutrition at the gene expression level in rice.

## Methods

### Aeration treatments

Seeds of the YD6 and NK57 rice cultivars were surface sterilized with 30 % (v : v) NaClO (5.2 % available chlorine) for 30 min and then rinsed thoroughly five or six times with water. The sterilized seeds were distributed uniformly on plastic mesh grates with 1-mm^2^ holes and placed in darkness for 2.5 days. The germinated seeds were divided equally into two groups for IA and EA treatments. Plastic grates containing germinated seeds for each group were placed on plastic buckets 30 cm in height and 25 cm in diameter. For the EA group, air was pumped continuously into the water in the bucket using an air pump. For seedlings in the IA group, air was not pumped into the water. The dissolved O_2_ level with IA and EA treatments was detected with a portable dissolved O_2_ detector (JPB-607, Leici Company, Shanghai, China) and recorded continuously during the 8 days of all experiments. Both groups of rice plants were placed in a growth chamber with a 16-h/8-h light/dark cycle and 30 °C/26 °C day/night temperature cycle. Relative humidity was maintained at 60 %. After 5 days of IA or EA treatment, the numbers of adventitious roots were counted and total root lengths and root surface areas were determined (scanned and analysed) using the WinRhizoV4.0b image analysis software (Regent Instruments, Canada). Root tips were collected for aerenchyma measurements and total root systems grown under the two aeration treatments were harvested 2 h into the light phase of the daily light cycle for analysis of *OsPAD4* (AK243523), *OsLSD1.1* (AK111759), *OsBphi008a* (NM_001048814), *OsPDCD5* (AY749430), *OsNRT2.1* (AB008519), *OsNAR2.1* (AP004023), *OsNRT2.3* (AK109776), *OsAMT1.1* (AF289477), *OsAMT1.2* (AF289479) and *OsAMT1.3* (AF289478) expression using quantitative real-time polymerase chain reaction (PCR). The shoots and roots of rice seedlings were separately collected for measuring biomass and total N. The samples were desiccated in a forced-air oven at 70 °C for ∼72 h to a constant weight, after which their dry weight was measured. The measurement of total N contents was performed according to Cai *et al*. (2012).

### Determination and quantification of aerenchyma formation

After IA and EA treatments, roots with lengths of 5–8 cm were selected for aerenchyma measurements. Root tissue was prepared as described previously (Ai *et al*. 2009) with minor modifications for the use of Spon812 embedding resin (SPI, USA) according to the manufacturer's specifications. Excised root segments were 1–1.5 cm (where aerenchyma development begins) or 2–2.5 cm (where aerenchyma development is completed) from the root tip. The sections were observed under a microscope (DM 5000 B, Leica, Germany), and aerenchyma formation was calculated from section images using ImageJ software.

### Nitrogen treatments

Rice seedlings of identical height cultivated under IA and EA conditions for 5 days were selected for N treatments for a further 2 days. Seedlings were divided into five groups for treatment under different N conditions. The seedlings were grown in tanks containing 10 L of nutrient solution. The five N treatments were listed as no N (−N), 0.125 mM NH_4_NO_3_ (LN), 1.25 mM Ca(NO_3_)_2_ (NO_3_-N), 1.25 mM (NH_4_)_2_SO_4_ (NH_4_-N) and 1.25 mM NH_4_NO_3_ (N/N). To inhibit nitrification, 7 µM dicyandiamide (DCD-C_2_H_4_N_4_) was added to the nutrient solutions. Whole roots were collected after 0.5, 2, 6, 12, 24 and 48 h of growth in the nutrient solutions to analyse gene expression. Shoots and roots of rice seedlings were, respectively, collected 48 h later to measure biomass and total N concentration. Plant samples were desiccated in a forced-air oven at 70 °C for ∼72 h to a constant weight, after which their dry weight was measured. The measurement of total N contents was performed according to Cai *et al*. (2012).

### Quantification of ethylene production from rice

The ethylene levels of five seedlings of the two rice varieties were determined after aeration and N treatments. Seedlings were transferred into 10 mL gas chromatography (GC) vials (B7990-6A, National Scientiﬁc Company, Rockwood, TN, USA). We then added 4 mL of the same N solution as used for the N treatments into IA group vials or waterman filter paper soaked with same N solutions into EA group vials and collected ethylene released from the seedlings for 12 h. The 6 mL total air in these vials was introduced into 50 mL syringes and subjected to GC analysis (GC7890, Agilent Company, Palo Alto, CA, USA) as described previously (Xu *et al*. 2008). Three replicates were used for the data pool.

### Quantitative real-time PCR

Total RNA was prepared from the roots of YD6 and NK57 seedlings using TRIzol reagent (Invitrogen; http://www.invitrogen.com). For quantitative PCR (qPCR) analysis, total RNAs were treated with DNaseI and reverse transcribed using SuperScript II (Invitrogen) to produce cDNA. Triplicate quantitative assays were performed on each cDNA dilution using SYBR Green Master Mix and an ABI 7000 sequence detection system according to the manufacturer's protocol (Applied Biosystems, http://www.takara.com). Gene-specific primers were designed using the Primer Express software (Applied Biosystems). The relative quantification method was used to evaluate the quantitative variation between replicates. Amplification of *OsRAc1* (Actin) was used as an internal standard to normalize all expression data. All primers used for qPCR are listed in **Supporting Information—Table S1**.

### Statistical analysis

All statistical evaluations were conducted using IBM SPSS ver. 13 software. To determine whether dissolved O_2_ in response to EA was changed during 192 h, a two-way analysis of variance (ANOVA) with aeration and time course as two main effects was used. One of the aims of the experiment was to investigate the nature of the aeration responses in rice plant. We measured adventitious root number, root surface area, aerenchyma formation, ethylene production and the expression of aerenchyma-formation- and N-related genes by aeration treatment of each cultivar individually. These data except gene expression were compared with the differences between cultivars under aeration treatment with two-way repeated-measures ANOVA. For the gene expression of each cultivar, one-way ANOVA was used to test significance of data difference. In order to study the interaction between aeration and N treatment on rice response, we used a full model factorial experiment ANOVA to test for main and interactive effects of cultivar, aeration and N treatments on rice dry weight, total N concentration and ethylene production. Furthermore, we investigated the influence of aeration during treatment time course on aerenchyma-formation- and N-usage-related gene expression in two rice varieties under LN and NO_3_-N conditions. The significant differences of gene expression over aeration time under different N conditions were determined by the analysis of covariance (ANCOVA).

## Results

### External aeration treatment increased root growth, and altered root aerenchyma formation and ethylene production under different N conditions

**Figure 1.**
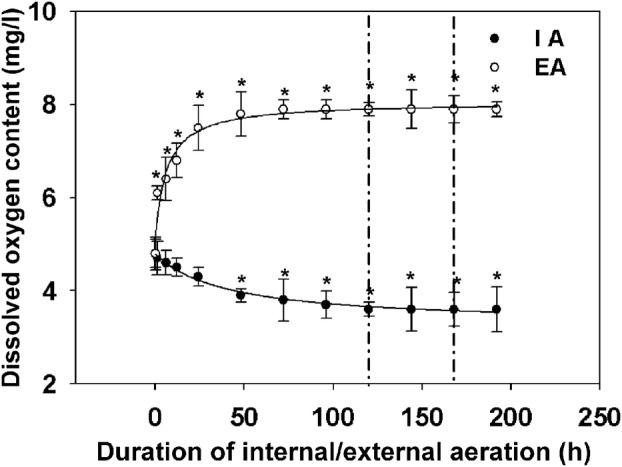
The dissolved O_2_ level in the solutions under IA and EA conditions. Measurements were continuous for 8 days in all experiments. IA, internal aeration (filled circles); EA, external aeration (open circles). The period between the two dashed lines indicates the time of N treatment. Significant differences relative to the dissolved O_2_ level at 0 h are indicated by asterisks (*P* < 0.05, two-way ANOVA).

To examine the effect of the dissolved O_2_ level on root growth, we quantified changes in the dissolved O_2_ level of the rice-growing solutions after EA. Under IA conditions, the dissolved O_2_ decreased from 4.3 to 3.8 mg L^−1^ by 48 h and stabilized at 3.6 mg L^−1^ after 120 h of growth (Fig. 1). Under EA conditions, the dissolved O_2_ increased from 4.8 to 6.8 mg L^−1^ by 48 h and reached 7.8 mg L^−1^ by 120 h (Fig. 1). Both IA and EA significantly affected the dissolved O_2_ in the solution (Table 1).

**Table 1.** Results from a two-way ANOVA evaluating the influence of aeration and duration on dissolved O_2_ content.

Source	df	Mean^2^	*F*	*P*
Aeration	1	302.419	3121.742	<0.0001
Duration	11	0.920	9.500	<0.0001
Aeration × duration	11	5.314	54.856	<0.0001
Error	96

**Figure 2.**
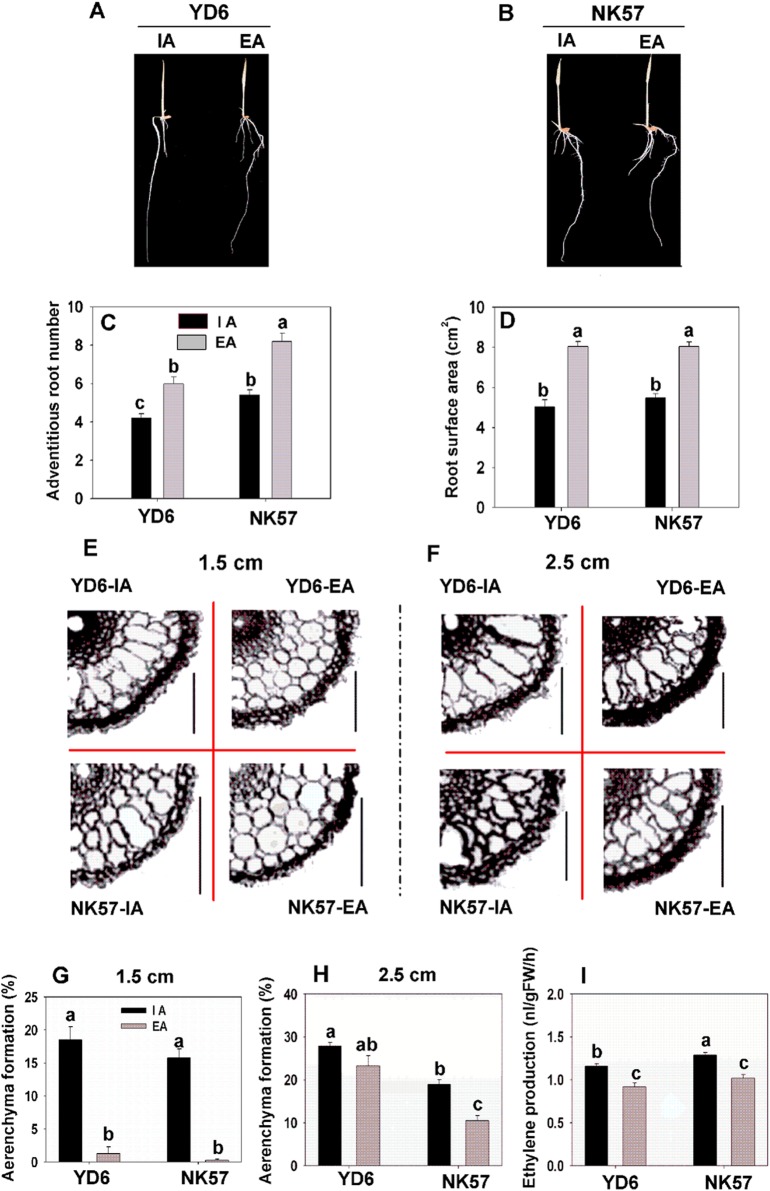
Rice growth, aerenchyma formation in root and ethylene production under IA and EA conditions. (A and B) Shoot and root phenotypes in seedlings of the YD6 (A) and NK57 (B) rice cultivars germinated and grown hydroponically in water for 5 days with IA or EA treatments. (C and D) Quantification of phenotypes. Numbers of adventitious roots (C) and root surface areas (D) were determined. (E and F) Partial aerenchyma formation visualized in sections of YD6 and NK57 roots. Resin-embedded sections obtained 1.5 cm (E) and 2.5 cm (F) from the root tips of YD6 and NK57 seedlings subjected to IA and EA treatments. (G and H) Quantification of aerenchyma formation in sections obtained 1.5 cm (G) and 2.5 cm (H) from the root tips, using Image J software. (I) Ethylene production in YD6 and NK57. Values represent the means ± SE (error bars) of three replicates. Significant differences are indicated by different letters (*P* < 0.05, two-way ANOVA).

The N treatments were started after 120 h when the dissolved O_2_ levels were stable. EA improved rice growth under different N supply conditions (Fig. 2A and B). Under conditions lacking N, both cultivars showed significant increases in the number of adventitious roots and root surface area (Fig. 2C and D) in response to EA treatment. While the number of adventitious roots showed significant higher in NK57 than YD6 at the same aeration condition (Fig. 2C and Table 2A), and the surface area had no significant difference (Fig. 2D and Table 2B) between cultivars. The shoot dry weight increased significantly in NK57, but not in YD6, after EA treatment (Table 3). In the treatments using different N supplies, EA treatment increased the shoot biomass by 15–40 % in NK57 and the root biomass by 20–50 % in both cultivars (Table 3). The results from a full model factorial experiment ANOVA of dry weight (Table 2) showed significant differences between cultivars, between aeration treatments and among N treatments (Table 4A). Furthermore, significant interactions were noted between cultivars and aeration, between aeration and N treatments, and between all three factors according to plant dry weight (Table 4A).

**Table 2.** Results from a two-way ANOVA evaluating the influence of aeration on adventitious root number, root surface area, aerenchyma formation and ethylene production of different rice seedlings.

Source	df	Mean^2^	*F*	*P*
(A) Adventitious root number				
Cultivar	1	14.450	34.000	<0.0001
Aeration	1	26.450	62.235	<0.0001
Cultivar × aeration	1	1.250	2.941	0.106
Error	16			
(B) Root surface area				
Cultivar	1	0.302	0.580	0.454
Aeration	1	48.245	92.773	<0.0001
Cultivar × aeration	1	0.333	0.641	0.432
Error	16			
(C) Aerenchyma formation (1.5 cm)				
Cultivar	1	12.592	8.885	0.018
Aeration	1	816.049	575.829	<0.0001
Cultivar × aeration	1	1.682	1.187	0.308
Error	8			
(D) Aerenchyma formation (2.5 cm)				
Cultivar	1	356.430	167.600	<0.0001
Aeration	1	125.453	58.991	<0.0001
Cultivar × aeration	1	11.213	5.273	0.051
Error	8			
(E) Ethylene production				
Cultivar	1	0.038	27.862	0.001
Aeration	1	0.191	140.355	<0.0001
Cultivar × aeration	1	0.001	0.674	0.435
Error	8

**Table 3.** Dry weights and total N of rice seedlings with different N supplies under IA and EA conditions. Seedlings were sampled under IA and EA conditions, with −N, LN, NO_3_-N, NH_4_-N and N/N added. Values represent the means ± SE (error bars). Letters associated with means correspond to the results of multiple comparison tests.

Treatment	Dry weight (g)							
	Shoot				Root			
	YD6		NK57		YD6		NK57	
	IA	EA	IA	EA	IA	EA	IA	EA
−N	3.4^b^	3.9^b^	3.8^b^	4.5^a^	1.8^c^	2.2^b^	1.9^c^	2.5^a^
LN	3.6^b^	4.1^b^	4.0^b^	4.9^a^	1.6^b^	2.3^a^	1.8^b^	2.6^a^
NO_3_-N	4.1^b^	4.4^b^	4.1^b^	5.1^a^	1.9^b^	2.7^a^	2.0^b^	2.7^a^
N/N	3.7^c^	4.2^b^	4.3^b^	5.0^a^	1.7^b^	2.3^a^	2.1^b^	2.6^a^
NH_4_-N	3.4^c^	4.2^b^	3.6^c^	5.4^a^	1.4^b^	2.3^a^	1.4^b^	2.7^a^
Treatment	Total N (mg g^−1^)							
	Shoot				Root			
	YD6		NK57		YD6		NK57	
	IA	EA	IA	EA	IA	EA	IA	EA
−N	38.0^a^	39.3^a^	37.2^a^	40.4^a^	14.2^c^	19.4^b^	22.2^a^	23.4^a^
LN	41.9^a^	43.1^a^	42.2^a^	43.7^a^	15.0^c^	23.2^b^	25.3^a^	25.0^a^
NO_3_-N	43.8^a^	43.4^a^	40.1^a^	42.4^a^	25.1^a^	25.0^a^	17.7^b^	25.3^a^
N/N	45.7^ab^	48.3^a^	42.9^b^	43.4^b^	26.1^a^	23.7^a^	25.1^a^	24.1^a^
NH_4_-N	48.3^a^	43.8^ab^	42.5^b^	46^b^	24.3^a^	23.3^a^	24.6^a^	25.0^a^

**Table 4.** Results from a full model factorial experiment ANOVA of dry weight, total N and ethylene production in rice seedlings with cultivar, aeration and N supplied conditions as factors.

Source	df	Mean^2^	*F*	*P*
(A) Dry weight				
Cultivar	1	8.817	93.208	<0.0001
Aeration	1	34.051	359.975	<0.0001
N	4	1.109	11.724	<0.0001
Cultivar × aeration	1	1.350	14.272	0.001
Cultivar × N	4	0.193	2.044	0.107
Aeration × N	4	0.832	8.799	<0.0001
Cultivar × aeration × N	4	0.172	1.815	0.145
Error	80			
(B) Total N				
Cultivar	1	29.506	18.568	<0.0001
Aeration	1	19.184	12.072	0.001
N	4	32.510	20.458	<0.0001
Cultivar × aeration	1	1.576	0.991	0.325
Cultivar × N	4	49.839	31.363	<0.0001
Aeration × N	4	17.035	10.720	<0.0001
Cultivar × aeration × N	3	37.267	23.451	<0.0001
Error	80			
(C) Ethylene production				
Cultivar	1	0.121	37.204	<0.0001
Aeration	1	0.590	182.018	<0.0001
N	4	0.041	12.761	<0.0001
Cultivar × aeration	1	0.010	3.048	0.088
Cultivar × N	4	0.002	0.713	0.588
Aeration × N	4	0.039	11.974	<0.0001
Cultivar × aeration × N	4	0.001	0.164	0.955
Error	40

To detect the effects of aeration on aerenchyma formation, root segments were sectioned at 1–1.5 and 2–2.5 cm from the root tip. Transverse root sections showed that aerenchyma was more developed in YD6 than in NK57, and that it was less well developed under EA conditions in both cultivars (Fig. 2E and F; **see** **Supporting Information—Fig. S1****A and B**). This effect was particularly noticeable in the sections obtained 1.5 cm from the root tip, in which aerenchyma formation was reduced from ∼16 to 0 % by EA in both cultivars (Fig. 2G; **see Supporting Information—Fig. S1C**). In the sections obtained 2.5 cm from the root tip, aerenchyma formation was decreased in NK57 roots by ∼9 % (Fig. 2H; **see** **Supporting Information—Fig. S1****D**). Aerenchyma formation was significantly different between cultivars and between aeration treatments but not between cultivar and aeration treatment (Table 2C and D).

**Figure 3.**
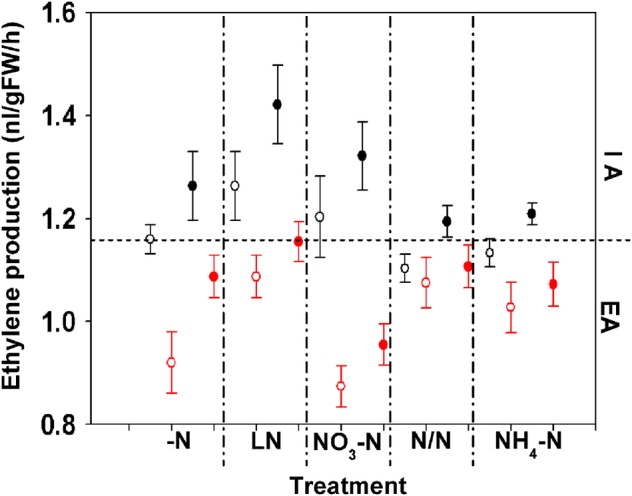
Ethylene production of YD6 and NK57 with different N supply. Black indicates ethylene production under IA conditions and red indicates ethylene production under EA conditions of YD6 (open circles) and NK57 (filled circles) seedlings with −N, LN, NO_3_-N, NH_4_-N and N/N treatments. Values represent the means ± SE (error bars) of three replicates.

Ethylene production in NK57 was higher than that in YD6 after IA treatment, but no difference was observed after EA (Fig. 2I). Under EA conditions, ethylene production in both cultivars was significantly lower than that under IA treatments without N added (Fig. 2I and Table 2E). Under different N supplied conditions, ethylene production showed significant differences between cultivars, aeration conditions and N treatments. There was also a significant interaction between aeration and N treatments (Fig. 3 and Table 4C). Furthermore, under IA conditions, LN and NO_3_-N enhanced ethylene production in both rice cultivars compared with N/N and $\text{N}{{\text{H}}_{4}}^{+}\text{- N}$ treatments (Fig. 3).

### External aeration treatment increased total N concentration in the roots under N-limited or nitrate-only conditions

Since root morphology and aerenchyma formation were both affected by EA treatment, and EA could affect N transport and acquisition in plants (Hu *et al*. 2014; Saengwilai *et al*. 2014), we further explored the effects of aeration on total N accumulation under varying N supply conditions. No significant differences were observed in the shoot N concentration in either cultivar in response to EA treatment (Table 3). In the roots of YD6 grown without N or in a LN solution, and in those of NK57 grown in a NO_3_-N solution, total N concentration increased significantly after EA treatment (Table 3). However, the total N concentration in the roots of both rice cultivars did not change significantly when grown under ammonium only or a mixed supply of nitrate and ammonium (Table 3). And the total N concentration showed no significant difference between cultivars, but there was a significant difference for aeration or N treatment (Table 4B).

### External aeration treatment affected the expression of aerenchyma-formation-related genes in roots, depending on the rice cultivar and N supply

**Figure 4.**
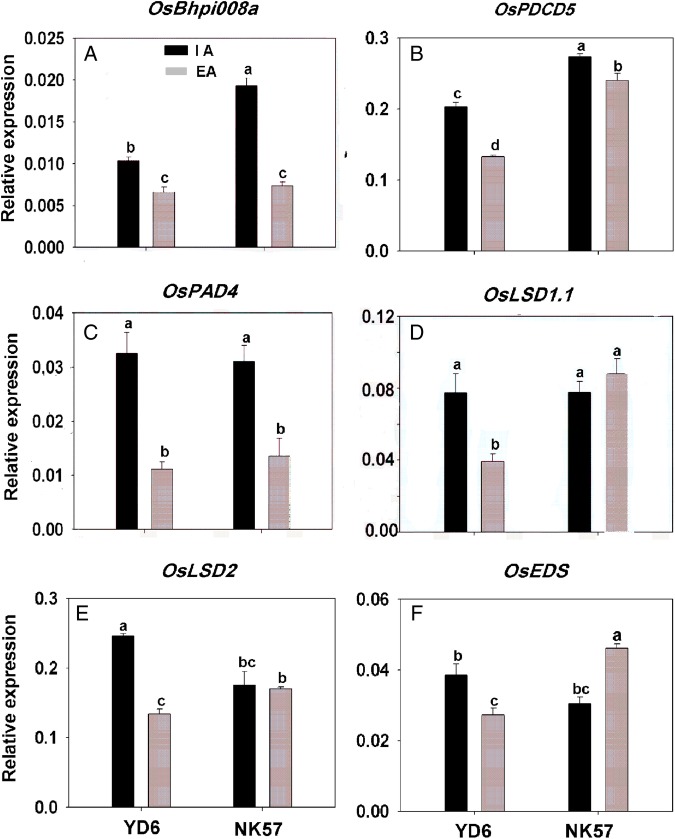
Real-time quantitative reverse transcription (RT)-PCR analysis of aerenchyma-formation-related gene expression in YD6 and NK57 roots under IA and EA conditions without N supply. RNA was extracted from 5-day-old seedlings grown hydroponically in water with aeration or non-aeration. Expression of *OsBphi008a* (A), *OsPDCD5* (B), *OsPAD4* (C), *OsLSD1.1* (D), *OsLSD2* (E) and *OsEDS* (F), which indicated ethylene content and aerenchyma formation, was examined. *OsActin* expression was used for normalization. Values represent the means ± SE (error bars) of three replicates. Significant differences are indicated by different letters (*P* < 0.05, one-way ANOVA).

**Figure 5.**
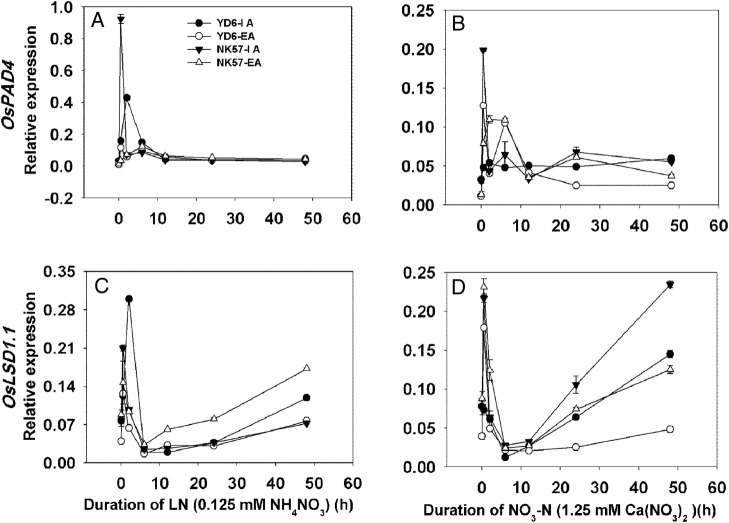
Real-time quantitative RT-PCR analysis of aerenchyma-formation-related gene expression in the roots of rice seedlings grown in LN and NO_3_-N nutrient solutions under IA and EA conditions. (A and B) Time course of *OsPAD4* expression under LN (A) and NO_3_-N (B) conditions. (C and D) Time course of *OsLSD1.1* expression under LN (C) and NO_3_-N (D) conditions. Seedlings grown with or without aeration were transferred to LN or NO_3_-N nutrient solution and roots were collected for gene expression analysis at 0, 0.5, 2, 6, 12, 24 and 48 h. Values represent means ± SE (error bars) of three replicates.

Since root aerenchyma formation was strongly affected by aeration treatment (Fig. 2), we determined the effects of aeration on the expression of aerenchyma-formation-related genes in roots. External aeration decreased the expression of the *OsBphi008a* and *OsPDCD5* genes by 12–43 % (Fig. 4A and B) and of *OsPAD4* by ∼60 % in both cultivars compared with IA treatment (Fig. 4C). Expression levels of *OsLSD1.1* (Fig. 4D), *OsLSD2* (Fig. 4E) and *OsEDS* (Fig. 4F) changed significantly after EA treatment in YD6 (downregulated by 58, 48 and 32 %, respectively). The expression of these genes in NK57 was not significantly different between the IA and EA treatments, except for *OsEDS*, which was upregulated (Fig. 4F). Nitrogen deficiency and NO_3_-N enhanced the porosity of, and aerenchyma formation in, rice roots (Wang *et al*. 2005; Abiko and Obara 2014). Therefore, a detailed time-course analysis of the expression was performed for aerenchyma-formation-related genes under different supplies of LN and NO_3_-N. The expression of *OsPAD4* and *OsLSD1.1* in YD6 peaked at 2 h under LN conditions (Fig. 5A and C) and at 0.5 h under NO_3_-N (Fig. 5B and D) conditions after IA treatment compared with EA treatment. After 48 h of aeration, the expression of *OsPAD4* was not affected in either rice variety compared with IA treatment under LN conditions (Fig. 5A and Table 5A). However, the expression of *OsLSD1.1* during 48 h of aeration showed the opposite trend in both rice varieties (Fig. 5C). *OsLSD1.1* expression was suppressed in YD6, but induced in NK57, by EA under LN conditions (Fig. 5C and Table 5B). Interestingly, the expression patterns of *OsPAD4* and *OsLSD1.1* under NO_3_-N supply differed from those under LN supply; expression levels of both *OsPAD4* (Fig. 5B) and *OsLSD1.1* (Fig. 5D) were significantly suppressed during aeration in both rice varieties (Table 5C and D). We also evaluated the expression of *OsPAD4* and *OsLSD1.1* under N/N and NH_4_-N conditions **[see** **Supporting Information—Fig. S2****]**. The results showed that *OsPAD4* expression was significantly downregulated in YD6 but significantly upregulated in NK57 by EA treatment with N/N **[see** **Supporting Information—Fig. S2****A]** and NH_4_-N **[see** **Supporting Information—Fig. S2****B]** supply.

**Table 5.** Results from an ANCOVA evaluating the influence of aeration and duration on aerenchyma-formation-related gene expression in different rice varieties under LN and NO_3_-N conditions.

Cultivar	Source	df	Mean^2^	*F*	*P*
(A) *OsPAD4* expression under LN conditions					
YD6	Duration	1	0.024	2.543	0.119
	Aeration	1	0.051	5.285	0.027
	Error	39			
NK57	Duration	1	0.153	3.223	0.080
	Aeration	1	0.142	2.983	0.092
	Error	39			
(B) *OsLSD1.1* expression under LN conditions					
YD6	Duration	1	0.010	2.094	0.156
	Aeration	1	0.020	4.153	0.048
	Error	39			
NK57	Duration	1	0.006	2.060	0.159
	Aeration	1	0.004	1.158	0.288
	Error	39			
(C) *OsPAD4* expression under NO_3_-N conditions					
YD6	Duration	1	<0.0001	0.385	0.538
	Aeration	1	<0.0001	0.264	0.610
	Error	39			
NK57	Duration	1	0.003	1.369	0.249
	Aeration	1	0.001	0.233	0.632
	Error	39			
(D) *OsLSD1.1* expression under NO_3_-N conditions					
YD6	Duration	1	0.001	0.594	0.446
	Aeration	1	0.001	0.544	0.465
	Error	39			
NK57	Duration	1	<0.0001	0.066	0.798
	Aeration	1	0.001	0.161	0.690
	Error	39

### External aeration treatment regulated the expression of several N transporter genes in the roots under different N conditions

**Figure 6.**
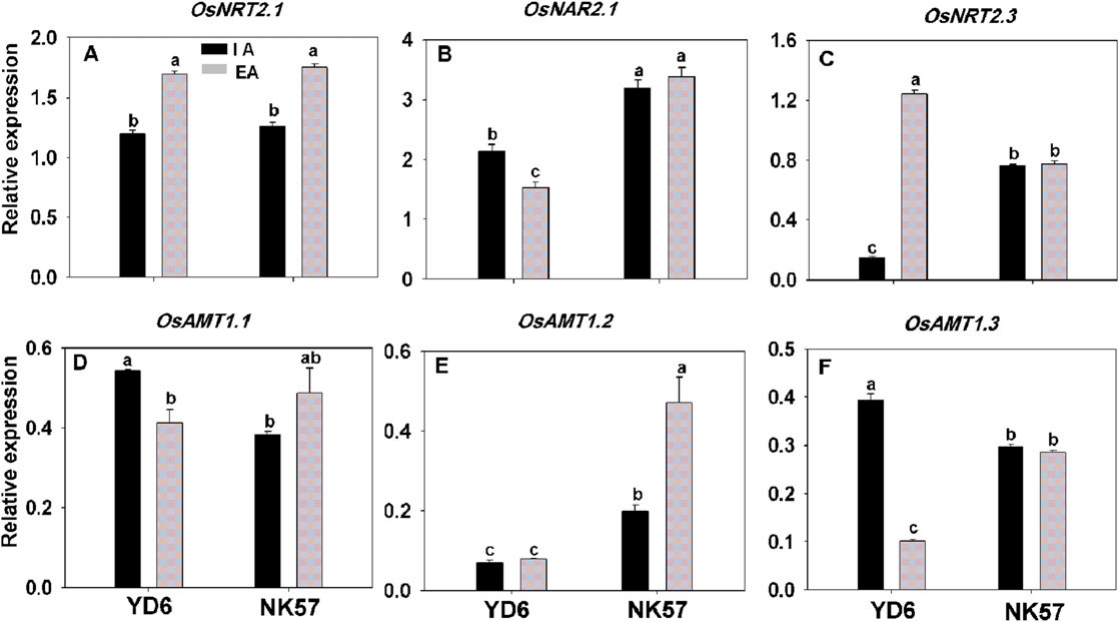
Real-time quantitative RT-PCR analysis of N transporter gene expression in YD6 and NK57 roots without N supply. RNA was extracted from 5-day-old seedlings grown hydroponically in water with IA or EA. (A–C) Expression of nitrate-uptake and -transporter genes. (D–F) Expression of ammonium transporter genes. *OsActin* gene expression was used for normalization. Values represent means ± SE (error bars) of three replicates. Significant differences are indicated by different letters (*P* < 0.05, one-way ANOVA).

**Figure 7.**
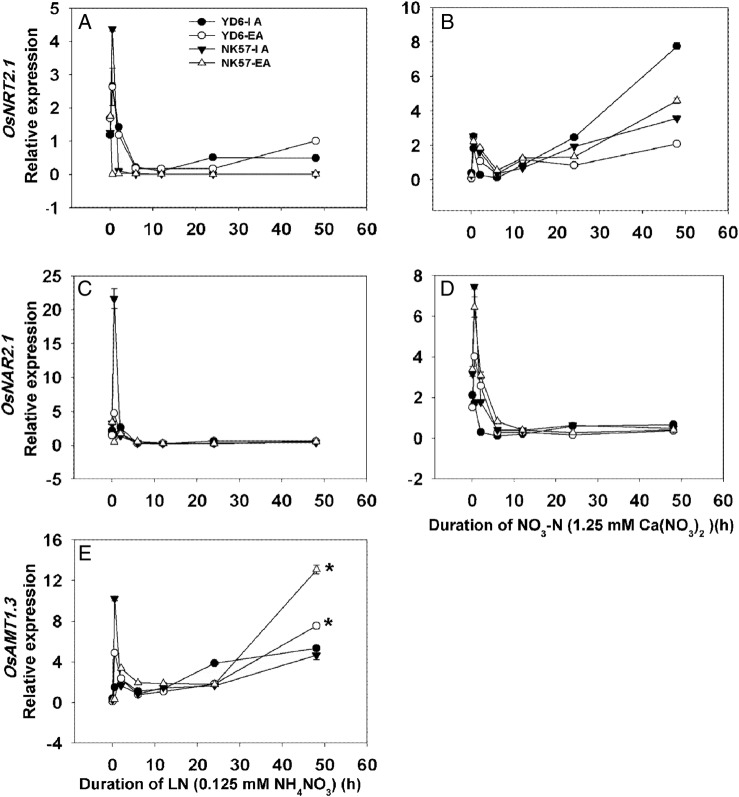
Real-time quantitative RT-PCR analysis of N-usage-related gene expression in the roots of rice seedlings grown in LN and NO_3_-N nutrient solutions with IA and EA treatments. (A and B) Time course of *OsNRT2.1* expression under LN (A) and NO_3_-N (B) conditions. (C and D) Time course of *OsNAR2.1* expression under LN (C) and NO_3_-N (D) conditions. (E) Time course of *OsAMT1.3* expression under LN conditions. Seedlings grown with or without aeration were transferred to a LN or NO_3_-N nutrient solution, and the roots were collected for gene expression analysis at 0, 0.5, 2, 6, 12, 24 and 48 h. Values represent the means ± SE (error bars) of three replicates.

Since EA increased the total N concentration in rice roots under limited N or NO_3_-N supply conditions (Table 3), we explored the effects of the N treatments on the expression of N transporter genes in rice seedlings grown with IA and EA treatments. The expression levels of the N-usage-related genes (*OsNRT2.1*, *OsNAR2.1*, *OsNRT2.3*; *OsAMT1.1*, *OsAMT1.2* and *OsAMT1.3*) were determined under −N supply. *OsNRT2.1* expression was increased by ∼30 % after EA treatment in both cultivars (Fig. 6A). *OsNAR2.1* (Fig. 6B) and *OsNRT2.3* (Fig. 6C) expression levels changed significantly after EA treatment in YD6 only. In YD6, *OsNAR2.1* (Fig. 6B) was downregulated by 31 %, but *OsNRT2.3* was upregulated (Fig. 6C). Interestingly, the expression of *OsNAR2.1* and *OsNRT2.3* in NK57 did not differ significantly between IA and EA treatments (Fig. 6B and C). The expression of *OsAMT1.1* (Fig. 6D) and *OsAMT1.3* (Fig. 6F), ammonium transporter genes, was significantly reduced by ∼20 and 75 % in YD6, respectively, while *OsAMT1.2* (Fig. 6E) expression was enhanced 2-fold in NK57 after EA treatment. It was also noted that expression of *OsNRT2.1*, *OsNAR2.1* and *OsAMT1.3* in YD6 and NK57 with or without EA changed over time in LN and NO_3_-N nutrient solutions (Fig. 7 and Table 6). Under LN conditions after 48 h of aeration, the expression levels of *OsNRT2.1* (Fig. 7A and Table 6A) and *OsAMT1.3* (Fig. 7E and Table 6B) increased in YD6; however, only *OsAMT1.3* was significantly upregulated in NK57 (Fig. 7E). Under NO_3_-N treatment, the expression of *OsNRT2.1* in YD6 was markedly downregulated by EA compared with IA (Fig. 7B and Table 6D). In NK57, the expression of *OsNRT2.1* after 48 h of aeration was increased by 40 % (Fig. 7B), while *OsNAR2.1* expression was not significantly different after 48 h of aeration under LN or NO_3_-N conditions (Fig. 7C and D and Table 6C and E).

**Table 6.** Results from an ANCOVA evaluating the influence of aeration and duration on N-usage-related gene expression in different rice varieties under LN and NO_3_-N conditions.

Cultivar	Source	df	Mean^2^	*F*	*P*
(A) *OsNRT2.1* expression under LN conditions					
YD6	Duration	1	13.176	27.213	<0.0001
	Aeration	1	0.042	0.088	0.769
	Error	39			
NK57	Duration	1	16.935	17.086	<0.0001
	Aeration	1	3.363	3.392	0.073
	Error	39			
(B) *OsAMT1.3* expression under LN conditions					
YD6	Duration	1	60.377	19.198	<0.0001
	Aeration	1	1.495	0.475	0.495
	Error	39			
NK57	Duration	1	68.054	5.189	0.028
	Aeration	1	0.702	0.054	0.818
	Error	39			
(C) *OsNAR2.1* expression under LN conditions					
YD6	Duration	1	26.407	27.197	<0.0001
	Aeration	1	0.276	0.284	0.597
	Error	39			
NK57	Duration	1	208.806	8.585	0.006
	Aeration	1	88.635	3.644	0.064
	Error	39			
(D) *OsNRT2.1* expression under NO_3_-N conditions					
YD6	Duration	1	37.915	13.750	0.001
	Aeration	1	6.627	2.403	0.129
	Error	39			
NK57	Duration	1	17.972	15.068	<0.0001
	Aeration	1	0.260	0.218	0.643
	Error	39			
(E) *OsNAR2.1* expression under NO_3_-N conditions					
YD6	Duration	1	21.900	28.819	<0.0001
	Aeration	1	2.602	3.424	0.072
	Error	39			
NK57	Duration	1	118.552	45.685	<0.0001
	Aeration	1	0.048	0.018	0.893
	Error	39

## Discussion

### External aeration treatment improved rice growth

Field oxygenation levels can affect rice growth and generally most rice cultivars show greater root biomass in deep-water or drained soils in comparison with conventional waterlogged growth conditions (Colmer *et al*. 2006; Parlanti *et al*. 2011). Oxygenation of hydroponic solutions used for rice growth increased adventitious root length, the absorption area and total biomass (Xu *et al*. 2012*a*). However, several reports have also shown that flooding treatments decrease adventitious root emergence and elongation (Visser and Pierik 2007; Dawood *et al*. 2014), and this was reviewed recently by Voesenek and Bailey-Serres (2015). In this study, we evaluated whether EA of the roots could improve rice plant growth and whether the response to O_2_ differed among rice cultivars (YD6 and NK57) with different aerenchyma formation. The number of adventitious roots, root surface area and root dry weight were increased by EA in YD6 and NK57 five days after germination (Fig. 2 and Table 3). The interaction between aeration and N on adventitious roots and biomass (Tables 2 and 4) indicated that O_2_ effects on rice plant growth depended on the N situation. Therefore, we deduced that the effect of O_2_ on adventitious root development of rice may depend on the different N conditions, since previous studies (Visser and Pierik 2007; Dawood *et al*. 2014) have focussed mainly on the effect of flooding on rice plants in potting soil conditions but not under specific N treatments. Although the effects of aeration on rice growth differed under different N conditions, EA treatment increased root dry mass more in the cultivar with less-developed aerenchyma (NK57) than in the cultivar with more-developed aerenchyma (YD6) (Table 3). However, the 48-h N treatments did not result in any significant differences in plant growth or N contents with different N treatments. This may be because the 48-h N treatment was too short to show any significant effects on rice growth.

### External aeration treatment decreased ethylene production and affected aerenchyma formation by regulating related gene expression

Root aerenchyma formation was increased by water flooding and inhibited by aeration or in drained soils (Colmer *et al*. 2006; Parlanti *et al*. 2011). Our results showed that aerenchyma formation and ethylene production in rice roots were reduced by EA treatment (Figs 2 and 3), and this is consistent with the findings from above. External aeration significantly inhibited ethylene production in two rice cultivars and decreased the formation of the root aerenchyma (Figs 2 and 3). The effect of ethylene on root aerenchyma formation was documented previously in rice (Nishiuchi *et al*. 2012). However, we found a significant interaction between aeration and N supply on rice ethylene production (Fig. 3 and Table 4). Under nitrate and −N conditions, the effects of IA and EA on rice ethylene production were much larger than those under ammonium or mixed N supply conditions (Fig. 3). This indicated that the ethylene signal in response to the external N supply appeared before the signal related to the biomass or N content (Fig. 3 and Tables 2 and 4). We also confirmed previous data showing that aerenchyma formation in different cultivars responded to external ethylene treatments (Justin and Armstrong 1991; Kong *et al*. 2009) using Tukey's test, which showed a significant difference between cultivars (Fig. 3 and Table 4). This indicated that the external ethylene treatment would be similar to the internal ethylene production, even under different N supply conditions. Surprisingly, the effect of aeration on ethylene production did not depend on the rice cultivar since the interaction between aeration and cultivar was not significant (Table 4). However, there was a significant interaction between aeration and N treatment, indicating that the external N effect on ethylene synthesis depends on the aeration conditions (Table 4). There was no interaction between cultivar and N treatment or between aeration and cultivar on ethylene production (Table 4).

Nitrogen deficiency and NO_3_-N treatments, compared with sufficient ammonium supply, enhance the porosity and formation of the aerenchyma in roots (Drew *et al*. 1989; He *et al*. 1994; Wang *et al*. 2005; Abiko and Obara 2014). Recently, Yang *et al*. (2012) reported that different sources of N nutrition affect aerenchyma formation in rice roots. However, the regulation of gene expression during aerenchyma formation in rice is unknown. In *Arabidopsis*, the *AtPAD4*, *AtLSD1* and *AtEDS1* genes play a role in ethylene synthesis and aerenchyma formation (Mühlenbock *et al*. 2007). *AtLSD1* is a negative regulator of PCD and plays a role in lysigenous aerenchyma formation. *AtPAD4* and *AtEDS* positively regulated the induction and amount of lysigenous aerenchyma formation, thereby counteracting the inhibitory action of *AtLSD1* (Mühlenbock *et al*. 2007). Based on these reports and our ethylene data, we explored the regulation of gene expression in rice roots by examining the expression of relevant genes, such as *OsBphi008a*, *OsPDCD5*, *OsPAD4* and *OsLSD1.1*.

In the aeration treatment experiment, aerenchyma formation was decreased in YD6 and NK57 roots (Fig. 2), and the PCD-related genes, *OsBphi008a* and *OsPDCD5*, were downregulated in roots (Fig. 4A and B). The expression pattern of *OsBphi008a*, which plays a positive role in ethylene synthesis (Hu *et al*. 2011), and *OsPDCD5*, which is involved in PCD (Drew *et al*. 2000), also exhibited positive regulation during rice aerenchyma formation, similar to the regulation pattern of ethylene and PCD in aerenchyma formation of *Arabidopsis* (Mühlenbock *et al.* 2007). Our results showed that *OsPAD4* expression was downregulated; the amount of root aerenchyma decreased in both rice cultivars, and *OsLSD1.1* expression was downregulated only in YD6 by EA (Fig. 4C and D). Therefore, we hypothesized that *OsPAD4* positively controlled aerenchyma formation in rice roots based on ethylene content, and that *OsLSD1.1* may be located upstream of *OsPAD4* in rice. In *Arabidopsis*, aeration and waterlogged treatments were performed under NO_3_-N conditions, and the expression of *AtPAD4* increased under waterlogging conditions (Mühlenbock *et al.* 2007). However, we compared the effect of aeration on *OsPAD4* under different N treatments, in which EA altered the expression of *OsPAD4* in a NO_3_-N solution (Fig. 5B) but not under low N conditions (Fig. 5A), a mixture of N or in a NH_4_-N solution **[see** **Supporting Information—Fig. S2****]**. The gene expression data showed that the ethylene-synthesis-related genes *OsPAD4* and *OsLSD1.1* peaked within 6 h of N treatment (Fig. 5). This indicates that the regulation of *OsPAD4* by EA was dependent on the external N supply and occurred during the very early stages of N treatment. Furthermore, the regulatory mechanism of aerenchyma formation during aeration in rice may differ from that in *Arabidopsis*.

### External aeration treatment increased rice N accumulation by increasing expression of the N transporter gene

Simultaneous supply of ammonium and nitrate improves plant growth, both in hydroponics and in soil (Xu *et al*. 1992; Qian *et al*. 2004; Ruan *et al*. 2007; Song *et al*. 2011). Most previous studies have reached this conclusion without considering the effect of O_2_ in the soil when applying different forms of N. In this study, it was shown that adding O_2_ through EA of the roots improved rice plant growth (Fig. 2) and N accumulation (Table 3), and that the N accumulation response to O_2_ differed among rice cultivars with different NUE. The results showed that total N concentration increased significantly by EA in YD6 roots without any supply of N (Table 3). Under low N conditions, the total N concentration increased in YD6 roots (Table 3), while in NK57 roots, NO_3_-N conditions with EA increased the total N concentration (Table 3). Tukey's test showed a significant interaction among the aeration conditions, cultivars and N treatment, which indicated that the external effect of N on different cultivar biomass and total N content depended on the aeration situation (Table 4).

The difference in N transporter gene expression between the two cultivars could explain the different N responses to EA treatments. The total N concentration in the roots of *OsNAR2.1* RNAi mutant plants was 63–66 % of that in wild-type roots grown in 0.2 mM N under IA conditions (Yan *et al*. 2011). In the *OsNAR2.1* RNAi mutant, the expression levels of *OsNRT2.1* and *OsNRT2.3a* decreased markedly (Yan *et al*. 2011). In the aeration experiment, the expression of *OsNRT2.1* and *OsNRT2.3* increased significantly in YD6 roots after 5 days of EA treatment; however, only *OsNRT2.1* expression was increased by EA treatment in NK57 roots (Fig. 6A–C). The gene expression data confirmed that the activities of enzymes involved in root N metabolism were enhanced by EA, and that the effect of EA on rice root N metabolism might be genotype specific (Xu *et al*. 2013). Under LN conditions, the expression levels of *OsNRT2.1* and *OsAMT1.3* were upregulated in YD6; however, expression of only *OsAMT1.3* was increased by EA in NK57 (Fig. 7). This difference in expression levels of *OsNRT2.1* (Fig. 7A) and *OsAMT1.3* (Fig. 7E) between the two cultivars may have caused the changes in N concentration in rice to EA under LN conditions (Table 3). However, under NO_3_-N supply conditions, the expression of a primary high-affinity transporter gene in rice roots (Araki and Hasegawa 2006), *OsNRT2.1*, was increased by EA in NK57 (Fig. 7B) but decreased in YD6 (Fig. 7B). The increase in *OsNRT2.1* expression may explain the increase in N concentration in NK57 in the presence of NO_3_-N condition with EA treatment. We conclude that the increased rice growth and total N acquisition by EA may be associated with the abundance of root endogenous aerenchyma and be dependent on the forms of N supplied.

## Conclusions

Following the aeration experiment, rice roots showed the different abundance of aerenchyma and growth pattern in YD6 and NK57 cultivars. The ethylene production from whole rice plant was also altered by aeration treatment in both cultivars. Combining aeration and external N treatments, we found a strong interaction effect between aeration and N supplies on rice growth and total N concentration in plant. Furthermore, the increase of rice growth and total N acquisition can be explained by altering expression of the *OsPAD4* and *OsNRT2.1* genes in aeration under LN and NO_3_-N supplies.

As in waterlogged conditions, the dissolved O_2_ was low and main N form was ammonium (Kludze *et al*. 1993; Wang *et al.* 1993). In contrast, in upland soil, the dissolved O_2_ was high and main N form was nitrate N (Kludze *et al*. 1993; Li *et al*. 2008). Apparently, the gene variation in responding to EA under different N conditions suggested that aeration and external N interaction were complex for plant adaptation to environmental change from waterlogged conditions into upland soil conditions. The EA could affect the formation of aerenchyma which in turn affected the growth of roots and shoots, and thus total N acquisition by altering expression of the *OsPAD4* and *OsNRT2.1* genes.

## Accession Numbers

Sequence data from this article can be found in the Rice Genome Initiative/GenBank data libraries under accession numbers listed in table **[see Supporting Information—Table S1]**.

## Sources of Funding

This research was funded by the National Natural Science Foundation (31172013) and PAPD (0306J0802).

## Contributions by the Authors

J.Z. and J.L. conducted all the experiments. Z.X. was involved in ethylene measurement. X.F. designed the experiments and wrote the manuscript. Q.Z. was involved in soluble O_2_ measurement. Q.S. and G.X. were involved in editing the manuscript.

## Conflict of Interest Statement

None declared.

## Supporting Information

The following additional information is available in the online version of this article –

**Table S1.** Primers and accession numbers used for quantitative real-time PCR.

**Figure S1.** Effects of aeration on aerenchyma formation of 7-day-old seedlings. Transverse section of root visualized in resin-embedded sections of YD6 (A) and NK57 (B) roots. Resin-embedded sections obtained 1.5 and 2.5 cm from the root tips of YD6 and NK57 seedlings subjected to IA and EA treatments. Quantification of aerenchyma formation in sections obtained 1.5 cm (C) and 2.5 cm (D) from the root tips, using Image J software. Values represent the means ± SE (error bars) of three replicates. Significant differences are indicated by different letters (*P* < 0.05, two-way ANOVA).

**Figure S2.** Real-time quantitative RT-PCR analysis of *OsPAD4* and *OsLSD1.1* expression in roots of rice seedlings grown in N/N and NH_4_-N nutrient solution. Time course of *OsPAD4* expression in N/N (A) and NH_4_-N (B) nutrient solution. Time course of *OsLSD1.1* expression in N/N (C) and NH_4_-N (D) nutrient solution. Seedlings grown with or without aeration were transferred to a LN nutrient solution and roots were collected for gene expression analysis at 0, 0.5, 2, 6, 12, 24 and 48 h. IA, internal aeration; EA, external aeration. Values represent the means ± SE (error bars) of three replicates.

Additional Information
