# Supplementary material for: Root aeration improves growth and nitrogen accumulation in rice seedlings under low nitrogen
Source: AoB Plants. 2015 Nov 17;7:plv131. doi: 10.1093/aobpla/plv131 (PMC4685170; doi:10.1093/aobpla/plv131)
Supplement: Additional Information [file supp_7_plv131_index.html]

Root aeration improves growth and nitrogen accumulation in rice seedlings under low nitrogen — Additional Information 

# Root aeration improves growth and nitrogen accumulation in rice seedlings under low nitrogen

## Additional Information

Additional Information

- Supplementary Table 1 - docx file
- Supplementary Figure 1 - docx file
