# Supplementary material for: Root aeration improves growth and nitrogen accumulation in rice seedlings under low nitrogen
Source: AoB Plants. 2015 Nov 17;7:plv131. doi: 10.1093/aobpla/plv131 (PMC4685170; doi:10.1093/aobpla/plv131)
Supplement: Additional Information [file supp_plv131_plv131supp_table1.docx]

**Supplemental Table S1. Primers used for quantitative real-time PCR**

| **Gene name and Gene ID** | **Primer** | **Sequence** |
| --- | --- | --- |
| ***OsLSD1.1***  **AK111759** | 5’（F） | GTGCAATGTTCTTGCTGTCACA |
|  | 5’（R） | CATGCGCAACTTGATTTGCT |
| ***OsEDS***  **AK100117** | 5’（F） | CCGCCGGTTGGTTGAG |
|  | 5’（R） | TCCTCGTTCTTGGAATGCCTAT |
| ***OsPAD***  **AK243523** | 5’（F） | GCATCAAGCTCGCACAGTTCT |
|  | 5’（R） | AGCTGGCTGGTGTCAAGCA |
| ***OsNAR2.1***  **AP004023** | 5’（F） | GTCGTCGAGAAGCGCAAGA |
|  | 5’（R） | GTCCACTGAAGCTGCGAACTT |
| ***OsNRT2.1***  **AB008519** | 5’（F） | CTTGTTGCAAACGGTGATGA |
|  | 5’（R） | GCCTCTCCCTTATTATACCTCCG |
| ***OsNRT2.3a***  **AK109776** | 5’（F） | GCCATCCACAAGATCGGTAG |
|  | 5’（R） | TGTGGAGCTTCCCGTAGTTG |
| ***OsAMT1.1***  **AF289477** | 5’（F） | AGCGAAGGAAGAAATCACG |
|  | 5’（R） | CCAAACAGAAACTGGCAATC |
| ***OsAMT1.2***  **AF289479** | 5’（F） | TTCTACGTGCTGCACAGGTTC |
|  | 5’（R） | TTGCTCCGGCGACTTTCT |
| ***OsAMT1.3***  **AF289478** | 5’（F） | GTCTAGTGGAACCGGAGGAG |
|  | 5’（R） | CCTATTATACAATCACGAAACCTG |
| ***OsBphi008a***  **NM_001048814** | 5’（F） | AGGATTACCATGGCCTCAAGAG |
|  | 5’（R） | CGCGTATCTTCCCCATGAAG |
| ***OsPDCD5***  **AY749430** | 5’（F） | TGGAGCAAATCAATACCCACACTA |
|  | 5’（R） | CTCCGGCGCCTCTGAAT |
