# Supplementary material for: Root aeration improves growth and nitrogen accumulation in rice seedlings under low nitrogen
Source: AoB Plants. 2015 Nov 17;7:plv131. doi: 10.1093/aobpla/plv131 (PMC4685170; doi:10.1093/aobpla/plv131)
Supplement: Additional Information [file supp_plv131_plv131supp_fig1.docx]

**

**

**Figure S1.** Effects of aeration on aerenchyma formation of 7-day-old seedlings. (A-B) Transverse section of root visualized in resin-embedded sections of YD6 (A) and NK57 (B) roots. Resin-embedded sections obtained 1.5 and 2.5 cm from the root tips of YD6 and NK57 seedlings subjected to internal aeration (IA) and external aeration (EA) treatments. (C-D) Quantification of aerenchyma formation in sections obtained 1.5 (C) and 2.5 (D) cm from the root tips, using the Image J software. Values represent the means ± SE (error bars) of three replicates. Significant differences are indicated by different letters (P < 0.05, two-way ANOVA).

**

**

**Figure S2.** Real-time quantitative RT-PCR analysis of *OsPAD4* and *OsLSD1.1* expression in roots of rice seedlings grown in 1.25 mM NH_4_NO_3_ (N/N) and 1.25 mM (NH_4_)_2_SO_4_ (NH_4_-N) nutrient solution. (A-B) Time course of *OsPAD4* expression in N/N (A) and NH_4_-N (B) nutrient solution. (C-D) Time course of *OsLSD1.1* expression in N/N (C) and NH_4_-N (D) nutrient solution. Seedlings grown with or without aeration were transferred to a LN nutrient solution and roots were collected for gene expression analysis at 0, 0.5, 2, 6, 12, 24 and 48 h. IA= internal aeration; EA= external aeration. Values represent the means ± SE (error bars) of three replicates.
